# Synthesis and Anti-Tumor Effects of Novel Pomalidomide Derivatives Containing Urea Moieties

**DOI:** 10.3390/ph15121479

**Published:** 2022-11-27

**Authors:** Yajie Guo, Xi Wang, Zhenzhen Wang, Longfei Mao, Jiahao Wang, Lizeng Peng, Guiqing Xu

**Affiliations:** 1 The Eighth Affiliated Hospital, Sun Yat-sen University, Shenzhen 518033, China; 2 School of Chemistry and Chemical Engineering, Henan Normal University, Xinxiang 453007, China; 3 State Key Laboratory of Medicinal Chemical Biology, College of Pharmacy and Tianjin Key Laboratory of Molecular Drug Research, Nankai University, Haihe Education Park, 38 Tongyan Road, Tianjin 300353, China; 4 Key Laboratory of Agro-Products Processing Technology of Shandong Province, Key Laboratory of Novel Food Resources Processing Ministry of Agriculture, Institute of Agro-Food Science and Technology Shandong Academy of Agricultural Sciences, Jinan 250100, China

**Keywords:** pomalidomide derivatives, urea moieties, immunomodulatory, anti-tumor effects

## Abstract

In order to explore novel immunomodulatory agents as anti-tumor drugs, we designed and synthesized a series of new pomalidomide derivatives containing urea moieties. Interestingly, in vitro biological experiments performed in several cancer cell lines showed that some of them displayed potent anti-tumor ability. These novel compounds **5a**–**5e** and **6a**–**6e** demonstrated the best cell growth inhibitive activity in human breast cancer cell lines MCF-7, but weaker inhibitive activity in human hepatocellular carcinoma cell lines Huh7. Moreover, compound **5d** had the most powerful effects in this study, with an IC_50_ value of 20.2 μM in MCF-7. Further study indicated that compound **5d** could inhibit cell growth and induce cell death in a concentration-dependent manner. Besides, compound **5d** increased cellular ROS levels and induced DNA damage, thereby potentially leading to cell apoptosis. These observations suggest that the novel pomalidomide derivatives containing urea moieties may be worth further investigation to generate potential anti-tumor drugs.

## 1. Introduction

**Figure 1:**
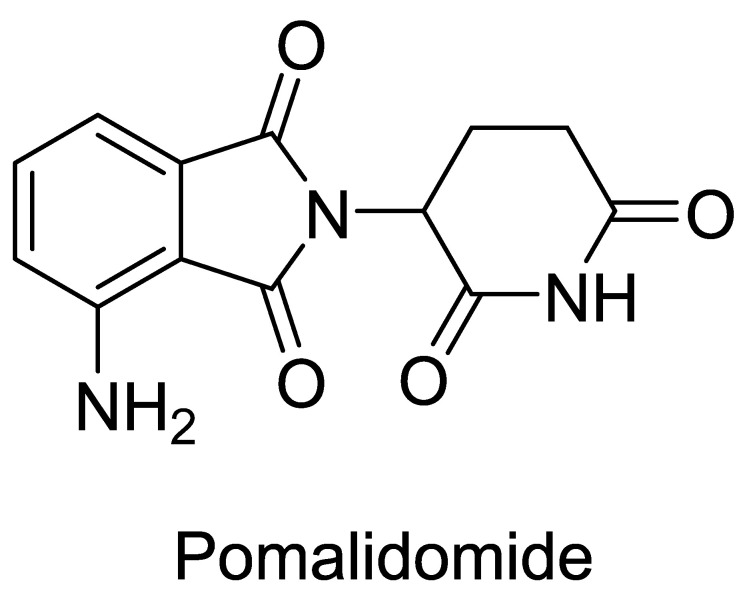
The structure of pomalidomide.

Immunomodulatory drugs (IMiD) are widely used in the clinical treatment of cancers. IMiD can stimulate T lymphocytes to increase IL-2 secretion and decrease the expression of proinflammatory cytokines [1,2]. Pomalidomide (Figure 1) is the third generation of IMiD produced by Celgene of the USA [3,4]. It is developed on the basis of the first generation of IMiD thalidomide, which modifies the molecular structure. The chemical name of pomalidomide is 4-amino-2-(2,6-dioxoperidine-3-ylisoindole-1,3-diketone). This improved IMiD can enhance the immune response mediated by T cells and NK cells, inhibit the production of monocyte proinflammatory cytokines, and induce apoptosis of cancer cells. Therefore, it is a popular treatment for various malignant tumors and immune diseases. Compared with the first and the second generation of IMiD, pomalidomide showed a stronger pharmacological effect, less toxicity, and better patient tolerance [5]. Because of its effective anti-angiogenesis and anti-inflammatory functions, pomalidomide is widely used in the early clinical studies of multiplemyeloma (MM) [6]. Recently, the molecular structure of pomalidomide has often been used as the ligand for E3 ligase in PROTAC production [7,8], and it plays an important role in the development of protease degradation drugs [9].

**Figure 2:**
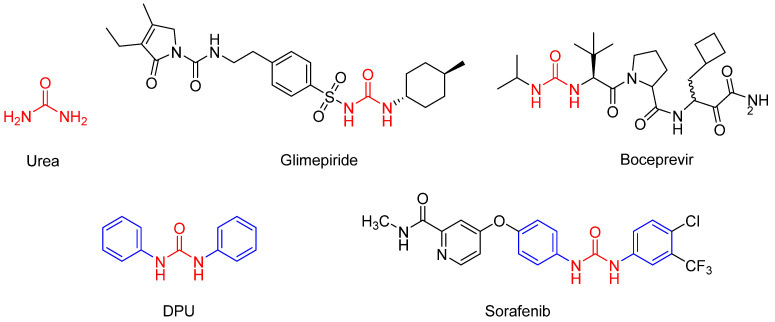
The structures of Urea, Glimepiride, Boceprevir, DPU, and Sorafenib.

The urea structure fragment (Figure 2) has a long and wide history in the application of medicinal chemistry. It consists of two aminos that are connected by carbonyl, and is an important fragment in drug development [10]. In the design of molecular drugs, the use of the urea structure fragment could improve activity, increase selectivity, and optimize physical and chemical properties; it is helpful for overcoming metabolic stability and removing toxic pharmacophore [11]. Until now, many drugs containing urea structure fragments that treat different diseases have been successfully marketed [12]. For example, glimepiride (Figure 2), a classic third generation oral hypoglycemic drug, is widely used in diabetes [13]. Boceprevir (Figure 2), an effective oral anti-hepatitis C drug, is widely used in hepatitis therapy [14]. N,N-diphenylurea (DPU) (Figure 2) is a kind of aromatic urea compound with a symmetrical structure and is widely found in plants with important roles. The function of DPU is similar to some cytokinin; it can promote chlorophyll synthesis and inhibit oxidase activity. DPU is a new plant growth regulator with important application values, which can help plants keep green, fresh, and delay aging. Moreover, derivatives of DPU are very important in medicine. Sorafenib (Figure 2), a multi-kinase inhibitor with N,N-diphenyl urea structure that can inhibit the activity of c-RAF, b-RAF, c-KIT, FLT3, PDGFR-α/β, and VEGFR-1/2/3 is usually used in tumor treatment, especially in acute myeloid leukemia (AML) clinical trials [15,16].

More and more studies focus on the therapeutic effects of novel synthesized compounds on tumors [17]. In order to explore more effective anti-tumor drugs, we linked pomalidomide and diphenyl urea compounds with different substituents through the ether chain structure and produced a series of novel pomalidomide derivatives containing urea moieties. Further study was performed to investigate the novel anti-tumor effects of these new compounds, include cell proliferation, apoptosis, ROS measurement, and molecular mechanisms. The aim of our study is to design and explore novel anti-tumor drugs that may provide potential strategy for cancers treatment.

## 2. Results and Discussion

### 2.1. Chemistry

**Scheme 1:**
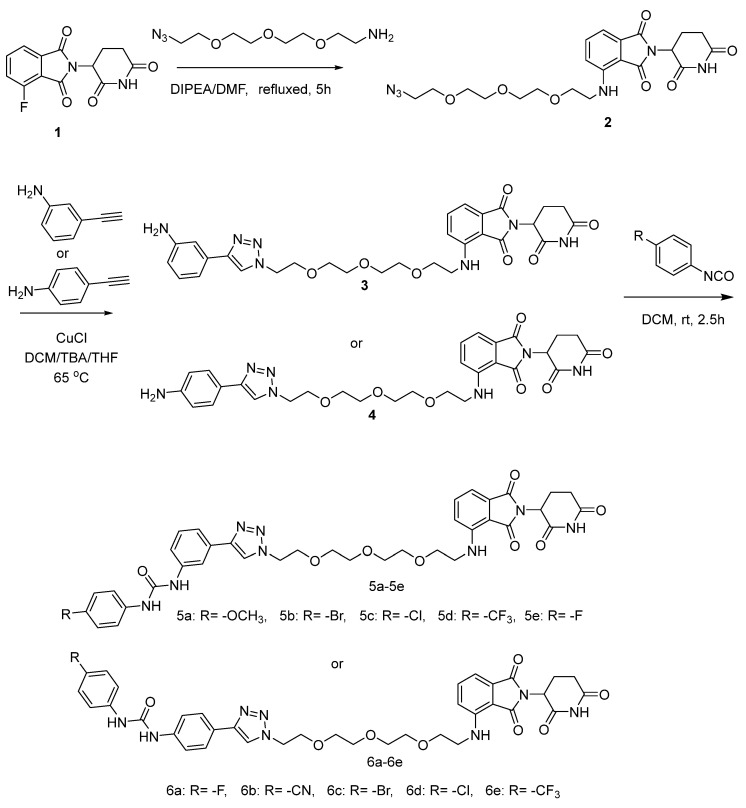
The reaction routes to compounds **5a**–**5e** and **6a**–**6e**.

The detailed operations are as follows (Scheme 1): The 2-(2,6-dioxo-piperidin-3-yl)-4-fluoro-isoindole-1,3-dione reacted with 2-(2-(2-(2-azidoethoxy)ethoxy)ethoxy)ethan-1-amine and DIPEA in the DMF to form compound **2**. Compound **2** reacted with 3-ethynylaniline or 4-ethynylaniline to form compound **3** or **4**. Compound **3** or **4** were then reacted with carbanil derivatives to give compound **5a**–**5e** or **6a**–**6e**. The molecular structures of these compounds were analyzed using ^1^H NMR (Supplementary Meterials), ^13^C NMR (Supplementary Meterials), elemental analyses, and MS.

### 2.2. Biology

#### 2.2.1. Effects of Novel Pomalidomide Derivatives Containing Urea Moieties on Cells Viability in Different Cancer Cell Lines

To investigate the effects of novel compounds (**5a**–**5e** and **6a**–**6e**) on cell toxicity and viability, we treated different cancer cell lines with different concentrations of these compounds for 48 h to calculate IC_50_. The chosen cell lines include human breast cancer cells MCF-7 and human hepatocellular carcinoma cells Huh7. The IC_50_ values of each compound in different cell lines were showed in Table 1. Results suggested that the cell growth inhibition activity of these compounds was better in MCF-7 cells than in Huh7 cells. Among these, compounds **5c** and **5d** showed more effective ability than others. The IC_50_ values of **5c** and **5d** in MCF-7 cells were 26.93 μM and 20.2 μM, respectively. Furthermore, we measured three control compounds’ IC_50_ values. These controls have similar structures with our new compounds. Con1 is 4-amino-2-(2,6-dioxopiperidin-3-yl) isoindoline-1,3-dione, con2 is 3-(4-amino-)-oxoisoindolin-2-yl) piperidine-2,6-dione, con3 is 2-(2,6-dioxopiperidin-3-yl) isoindoline-1,3-dione. The IC_50_ values of these controls were all higher than 200 μM. This suggested that the inhibitory effects against cancer cells of our modified compounds were significantly better than those of the controls. The data of IC_50_ showed that the inhibition activity of compounds **5a**–**5e** was better than **6a**–**6e**, this suggested a urea structure that, at the meta-position of phenyl triazole structure, had better biological activity, and trifluoromethyl was found to be the most active substituent.

#### 2.2.2. Novel Compound **5d** Suppressed Cancer Cell Proliferation

**Figure 3:**
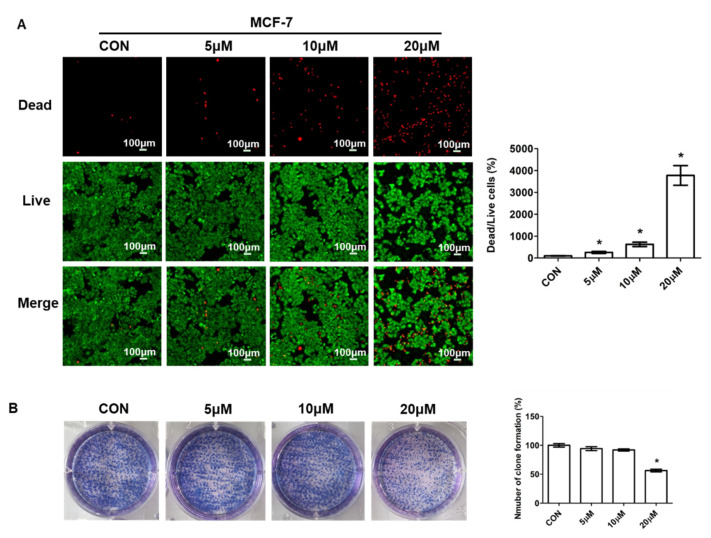
Compound **5d** suppressed cell proliferation. (**A**) MCF-7 cells were treated with 5 μM, 10 μM, 20 μM of compound **5d** for 24 h, then, cells were stained with live/dead dyes. Left: fluorescence confocal images; right: ratio of dead to live cells. (**B**) MCF-7 cells were treated with 5 μM, 10 μM, 20 μM of compound **5d** for 48 h, then, cells were cultured continuously until single cell clone appeared, followed by staining with Giemsa dye. Left: photographs of cells; right: statistical graph. Data were presented as means ± SEM, * *p* < 0.05.

The above results suggested that novel compounds, especially **5d**, had an effective anti-proliferative activity to cancer cells. To further evaluate the role of compound **5d** in cell proliferation, we performed the LIVE/DEAD staining and plate clone formation assay in MCF-7 cells. Cells were treated with different concentrations of compound **5d** for 24 h, then, live and dead cells were stained with different dyes and photographed. The living cells were stained in green and the dead cells were stained in red. As shown, the number of dead cells increased in a dose dependent manner after treatment (Figure 3A). The number of living cells was largely decreased in the 20 μM dose group compared with the control group (Figure 3A). The ratio of dead to live cells was also dramatically increased in all treated groups, and was affected by dosage (Figure 3A). Additionally, plate clone formation assay was detected to further investigate the anti-proliferation ability of compound **5d**. The results showed that cell proliferation activity was suppressed and it was also changed in a dose-dependent manner (Figure 3B).

#### 2.2.3. Novel Compound **5d** Induced Cell Apoptosis

**Figure 4:**
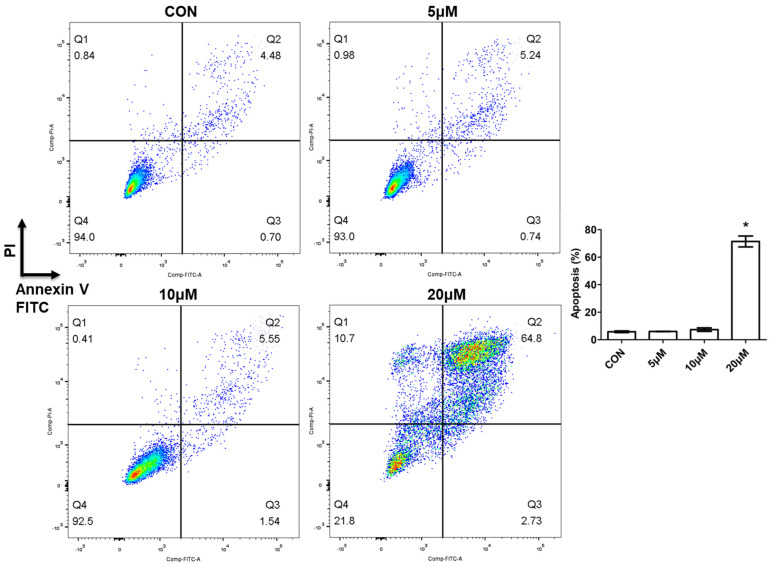
Compound **5d** induced cell apoptosis. MCF-7 cells were treated with 5 μM, 10 μM, 20 μM of compound **5d** for 48 h, then cells were collected and stained with Annexin-V and PI. Apoptotic cells were determined by flow cytometry. Data were presented as means ± SEM, * *p* < 0.05.

The above data showed that compound **5d** could inhibit cancer cell growth. To explore whether it had effects on cell apoptosis, we performed apoptosis analysis. After treatment with compound **5d** for 48 h, MCF-7 cells were collected and stained with Annexin-V and PI. Quadrant Q1 represents necrotic cells, quadrant Q2 represents late apoptotic cells, quadrant Q3 represents early apoptotic cells, and quadrant Q4 represents non-apoptotic cells. The results showed that cell apoptosis, especially late apoptosis, was significantly induced in the 20 μM group but did not change in the 5 μM or 10 μM group compared with the control group (Figure 4). Cell apoptosis is a continuous process that can occur at any phase of the cell cycle, and it is hard to define the exact phase of the cell at which apoptosis occurs.

#### 2.2.4. Novel Compound **5d** Increased ROS Levels and Induced DNA Damage

**Figure 5:**
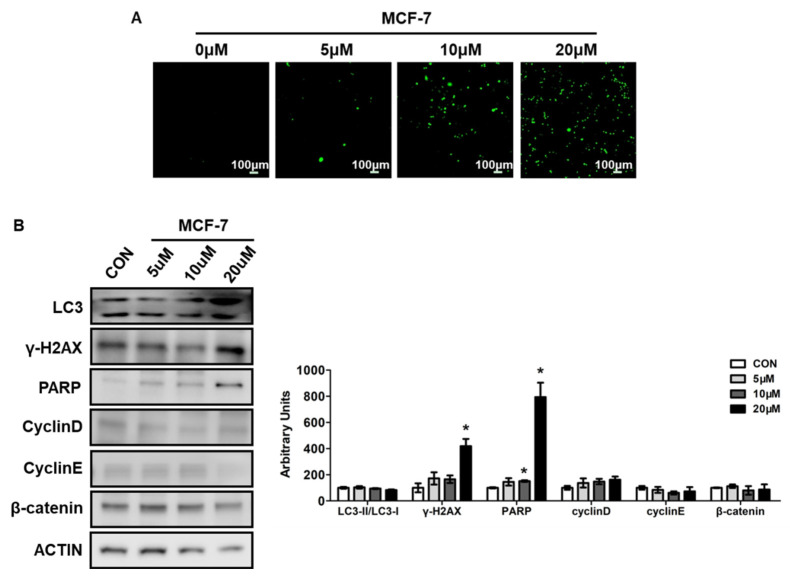
Compound **5d** increased ROS levels and induced DNA damage. (**A**) MCF-7 cells were treated with 5 μM, 10 μM, 20 μM of compound **5d** for 24 h, then cells were stained by DCFH-DA for 30 min, ROS were detected using fluorescent confocal microscope. (**B**) MCF-7 cells were treated with 5 μM, 10 μM, 20 μM of compound **5d** for 48 h, cell protein were collected and analyzed by Western boltting. Left: western bolt of LC3, γ-H2AX, PARP, Cyclin D, Cyclin E, β-catenin; right: quantitative measurements relative to ACTIN. Data were presented as means ± SEM, * *p* < 0.05.

Cellular reactive oxygen species (ROS) are mainly produced by the mitochondrial and NAPDH oxidase family, which includes superoxide anion (O_2_−), hydroxyl (OH−), and hydrogen peroxide (H_2_O_2_). ROS levels affect many intracellular signaling transductions and, thus, play very important roles in life processes [18]. The homeostasis of ROS is associated with cell growth and survival. Studies showed that in many cancers, ROS levels were obviously increased, which could affect genome stability, excessive proliferation, angiogenesis, and epithelial mesenchymal transformation [19]. Researchers found that appropriately increased ROS levels could stimulate tumor cell proliferation, but excessively increased ROS levels would lead to tumor cell apoptosis and death [20]. In order to investigate whether the apoptosis induced by compound **5d** was related to ROS levels, we treated MCF-7 cells with different doses of compound **5d** for 24 h and detected cellular ROS levels using a DCFH-DA probe. ROS levels were dramatically increased in all treated groups, and they increased as the concentration increased (Figure 5A).

Cell death can be caused by various processes, such as apoptosis, autophagy, and DNA damage [21]. To further evaluate these signaling pathways, key regulated protein expressions related to these processes were determined. Ubiquitin-like molecule light chain 3 (LC3) is the marker of autophagy, and results showed that the ratio of LC3II to LC3I did not change after compound **5d** treatment, although the expression of LC3I and LC3II was increased (Figure 5B). This suggests that autophagy may not be the main reason for cell death induced by compound **5d**.

DNA damage is another important cause of cell death and it was reported that proteins involved in DNA damage are also related to cell apoptosis [21]. After treatment with compound **5d**, the protein expressions involved in the DNA damage process increased, including H2AX variant histone (γ-H2AX) and poly (ADP-ribose) polymerase (PARP) (Figure 5B). Moreover, the cell cycle process was determined and did not change, as reflected by the unchanged expressions of CyclinD, CyclinE, or β-catenin (Figure 5B).

## 3. Materials and Methods

### 3.1. Materials and Chemistry

The 2-(2,6-dioxopiperidin-3-yl)-4-fluoroisoindoline-1,3-dione and 2-(2-(2-(2-azidoethoxy)ethoxy)ethoxy)ethan-1-amine were purchased from Sihma-Aldrich (St. Louis, MO, USA). The other reagents and solvents were obtained from Aladdin’s reagent (Shanghai, China). ^1^H NMR and ^13^C NMR spectra were acquired in DMSO-*d*_6_ solution with a Bruker400 spectrometer. Chemical shifts (d) were given in parts per million with tetramethylsilane as the internal reference and coupling constants were expressed in hertz. s (singlet), d (doublet), t (triplet), m (multiplet). Elemental analyses were conducted using an Elementar Analysensystem Vario Macro cube. CHNSO mode.

Dulbecco’s modified Eagle medium (DMEM), Fetal bovine serum (FBS) and penicillin/streptomycin were obtained from Gibco (Grand Island, NY, USA). Enhanced Cell Counting Kit-8, Calcein/PI Live/Dead Viability Assay Kit, Giemsa dye, Reactive Oxygen Species (ROS) Assay Kit, and Annexin V-FITC/ Propidium iodide (PI) staining kit were bought from Beyotime Biotechnology (Shanghai, China). Antibodies used in Western Blotting were: LC3, cyclin D, cyclin E, β-Catenin, γH2AX, PARP (all from Cell Signaling Technology, Danvers, MA, USA), β-actin (Sigma, Shanghai, China).

### 3.2. General Procedure for Preparation of Compound ***2***

2-(2,6-dioxopiperidin-3-yl)-4-fluoroisoindoline-1,3-dione (2.8 g, 0.01 mol) was added into dimethyl formamide (150 mL), then mixed with 2-(2-(2-(2-azidoethoxy)ethoxy)ethoxy)ethan-1-amine (2.2 g, 0.01 mol) and DIPEA (2.5 g, 0.02 mol), and placed in N2 atmosphere. The reaction mixture was refluxed for 5 h. After completion of the reaction, the mixture was washing with distilled water (100 mL) and extracted with CH_2_Cl_2_ (3 × 70 mL). The combined organic phase was successively washed with water and brine, dried over Na_2_SO_4_, and concentrated in vacuo to obtain 4-((2-(2-(2-(2-azidoethoxy)ethoxy)ethoxy)ethyl)amino)-2-(2,6-dioxopiperidin-3-yl)isoindoline-1,3-dione (compound **2**) as a pale yellow solid (2.9 g) [22].

### 3.3. General Procedure for the Synthesis of Compound ***3*** (The Method Is Suitable for Compound ***4***)

Compound **2** (4.8 g, 0.01 mol) and 3-ethynylaniline (1.4 g, 0.012 mol) were added to a mixed solvent (water: CH_2_Cl_2_: tert-butanol: THF = 1:1:1:1, 40 mL). Cuprous chloride (0.2 g, 0.002 mol) was added to the mixture and the reaction was stirred at 65 °C. After completion of the reaction, the mixture was extracted with CH_2_Cl_2_ (15 mL × 4). The combined organic phase was washed successively with water and brine, then drying with MgSO_4_ and desolventizing. The residue was purified by column chromatography (CH_2_Cl_2_/MeOH = 20:1) to obtain 4-((2-(2-(2-(2-(4-(3-aminophenyl)-1H-1,2,3-triazol-1-yl)ethoxy)ethoxy)ethoxy)ethyl)amino)-2-(2,6-dioxopiperidin-3-yl)isoindoline-1,3-dione (compound **3**) as a crystalline powder. ^1^H NMR (400 MHz, DMSO-*d*_6_) δ 11.10 (s, 1H), 8.33 (s, 1H), 7.11–7.03 (m, 4H), 6.92 (d, *J* = 8.0 Hz, 1H), 6.57 (t, 1*J* = 8.0 Hz, 2*J* = 4.0 Hz, 2H), 6.52 (d, *J* = 8.0 Hz, 1H), 5.16–5.03 (m, 3H), 4.53 (t, 1*J* = 4.0 Hz, 2*J* = 4.0 Hz, 1H), 3.84 (t, 1*J* = 4.0 Hz, 2*J* = 4.0 Hz, 2H), 3.58–3.50 (m, 7H), 3.44–3.40 (m, 2H), 2.93–2.84 (m, 1H), 2.61–2.53 (m, 1H), 2.04–2.01 (m, 1H). ^13^C NMR (100 MHz, DMSO-*d*_6_) δ 173.29, 170.56, 169.41, 167.76, 149.50, 147.34, 146.86, 136.70, 132.55, 131.76, 129.78, 121.74, 117.91, 113.97, 113.45, 111.14, 110.89, 109.70, 70.23, 70.21, 70.15, 70.09, 69.30, 69.13, 49.94, 49.02, 42.13, 40.54, 31.45, 22.61.

### 3.4. General Procedure for the Preparation of Compound ***5a*** (The Method Is Suitable for ***5b***~***5e***, ***6a***–***6e***)

A solution of compound **3** (6.0 g, 0.01 mol) in CH_2_Cl_2_ (100 mL) was added with 1-isocyanato-4-methoxybenzene (1.5 g, 0.01 mol) in one portion. After stirring at room temperature for 2.5 h, the mixture was concentrated, and the residue was purified by column chromatography on silica gel (eluent: PE: EA = 2:1) to give compound **5a** as a pale yellow solid (3.9 g).

1-(3-(1-(2-(2-(2-(2-((2-(2,6-dioxopiperidin-3-yl)-1,3-dioxoisoindolin-4-yl)amino)ethoxy)ethoxy)ethoxy)ethyl)-1H-1,2,3-triazol-4-yl)phenyl)-3-(4-methoxyphenyl)urea (Compound **5a**): Yield 72.5%; ^1^H NMR (400 MHz, DMSO-*d*_6_) δ 10.99 (s, 1H, NH-H), 8.58 (s, 1H, NH-H), 8.35 (d, *J* = 8.0 Hz, 1H, NH-H), 7.42 (t, 1*J* = 4.0 Hz, 2*J* = 8.0 Hz, 1H, Ar-H), 7.27–7.18 (m, 5H, Ar-H), 6.96–6.89 (m, 2H, Ar-H), 6.75 (d, *J* = 12.0 Hz, 2H, Ar-H), 6.46–6.42 (m, 1H, Ar-H), 5.62 (s, 1H, CH-H), 4.94 (dd, 1*J* = 8.0 Hz, 2*J* = 4.0 Hz, 1H, CH_2_-H), 4.45–4.40 (m, 2H, CH_2_-H), 3.74 (t, 1*J* = 4.0 Hz, 2*J* = 4.0 Hz, 2H, CH_2_-H), 3.60 (s, 3H, OCH_3_-H), 3.45–3.41 (m, 7H, CH_2_-H), 3.29–3.26 (m, 4H, CH_2_-H), 2.82–2.72 (m, 1H, CH_2_-H), 2.46–2.38 (m, 2H, CH_2_-H), 1.91 (d, *J* = 8.0 Hz, 1H, CH_2_-H). ^13^C NMR (100 MHz, DMSO-*d*_6_) δ 173.29, 170.57, 169.42, 167.77, 155.00, 153.21, 146.82, 146.75, 140.92, 136.62, 133.13, 132.53, 131.80, 129.78, 122.19, 121.73, 120.57, 120.40, 119.14, 118.01, 117.80, 115.14, 114.44, 111.13, 110.93, 109.71, 70.23, 70.16, 70.11, 69.28, 69.12, 55.62, 55.36, 50.04, 49.04, 42.12, 31.47, 22.63. MS (ESI) m/z: 741 [M+H]^+^. Anal. Calcd for C_37_H_40_N_8_O_9_: C, 59.99; H, 5.44; N, 15.13. Found: C, 59.76; H, 5.34; N, 15.27.

1-(4-bromophenyl)-3-(3-(1-(2-(2-(2-(2-((2-(2,6-dioxopiperidin-3-yl)-1,3-dioxoisoindolin-4-yl)amino)ethoxy)ethoxy)ethoxy)ethyl)-1H-1,2,3-triazol-4-yl)phenyl)urea (Compound **5b**): Yield 79.7%; ^1^H NMR (400 MHz, DMSO-*d*_6_) δ 11.14 (s, 1H), 8.86 (s, 2H), 8.50 (s, 1H), 8.07 (s, 1H), 7.57 (t, 1*J* = 8.0 Hz, 2*J* = 8.0 Hz, 1H), 7.49–7.45 (m, 5H), 7.42–7.35 (m, 2H), 7.11–7.05 (m, 2H), 6.59 (s, 1H), 5.09 (dd, 1*J* = 4.0 Hz, 2*J* = 4.0 Hz, 1H), 4.59 (s, 2H), 3.89 (t, 1*J* = 4.0 Hz, 2*J* = 4.0 Hz, 2H), 3.60–3.53 (m, 10H), 3.44 (s, 2H), 2.96–2.87 (m, 1H), 2.62–2.52 (m, 2H), 2.07–2.02 (m, 1H). ^13^C NMR (100 MHz, DMSO-*d*_6_) δ 173.29, 170.56, 169.40, 167.76, 152.88, 146.82, 146.66, 140.51, 139.56, 139.45, 136.64, 132.53, 131.98, 131.84, 129.83, 122.24, 120.65, 119.50, 118.23, 117.81, 115.36, 113.75, 111.13, 109.70, 70.22, 70.15, 70.10, 69.28, 69.10, 60.24, 55.36, 50.04, 49.03, 42.12, 31.46, 22.63, 21.22. MS (ESI) *m*/*z*: 789 [M+H]^+^. Anal. Calcd for C_36_H_37_BrN_8_O_8_: C, 54.76; H, 4.72; N, 14.19. Found: C, 54.59; H, 4.83; N, 14.02.

1-(4-chlorophenyl)-3-(3-(1-(2-(2-(2-(2-((2-(2,6-dioxopiperidin-3-yl)-1,3-dioxoisoindolin-4-yl)amino)ethoxy)ethoxy)ethoxy)ethyl)-1H-1,2,3-triazol-4-yl)phenyl)urea (Compound **5c**): Yield 78.3%; ^1^H NMR (400 MHz, DMSO-*d*_6_) δ 11.09 (s, 1H), 8.46 (s, 1H), 8.04 (s, 1H), 7.57–7.53 (m, 4H), 7.41–7.36 (m, 2H), 7.32–7.30 (m, 4H), 7.08 (d, *J* = 12.0 Hz, 1H), 7.02 (d, *J* = 8.0 Hz, 1H), 6.55 (s, 1H), 5.04 (dd, 1*J* = 4.0 Hz, 2*J* = 4.0 Hz, 1H), 4.55 (t, 1*J* = 4.0 Hz, 2*J* = 4.0 Hz, 2H), 4.04–4.00 (m, 3H), 3.85 (t, 1*J* = 8.0 Hz, 2*J* = 4.0 Hz, 3H), 3.56–3.52 (m, 9H), 2.89–2.83 (m, 1H), 2.60–2.56 (m, 2H), 2.03–2.00 (m, 1H). ^13^C NMR (100 MHz, DMSO-*d*_6_): δ 173.28, 170.56, 167.76, 153.17, 146.84, 146.76, 144.67, 139.55, 136.66, 132.54, 129.71, 128.98, 122.20, 120.19, 120.18, 118.22, 117.86, 115.37, 111.12, 109.69, 73.61, 70.66, 70.21, 70.15, 70.10, 69.28, 69.10, 63.82, 50.01, 49.02, 42.11, 31.78, 31.45, 22.60. MS (ESI) m/z: 745 [M+H]^+^. Anal. Calcd for C_36_H_37_ClN_8_O_8_: C, 58.02; H, 5.00; N, 15.04. Found: C, 57.94; H, 5.07; N, 15.15.

1-(3-(1-(2-(2-(2-(2-((2-(2,6-dioxopiperidin-3-yl)-1,3-dioxoisoindolin-4-yl)amino)ethoxy)ethoxy)ethoxy)ethyl)-1H-1,2,3-triazol-4-yl)phenyl)-3-(4-(trifluoromethyl)phenyl)urea (Compound **5d**): ^1^H NMR (400 MHz, DMSO-*d*_6_): 11.11 (s, 1H), 9.12 (s, 1H), 8.93 (s, 1H), 8.49 (s, 1H), 8.05 (s, 1H), 7.66 (dd, 1*J* = 8.0 Hz, 2*J* = 8.0 Hz, 4H), 7.55 (t, 1*J* = 4.0 Hz, 2*J* = 8.0 Hz, 1H), 7.44 (d, *J* = 4.0 Hz, 1H), 7.39–7.33 (m, 2H), 7.08 (d, *J* = 8.0 Hz, 1H), 7.02 (d, *J* = 8.0 Hz, 1H), 6.56(t, 1*J* = 4.0 Hz, 2*J* = 4.0 Hz, 1H), 5.05 (dd, 1*J* = 4.0 Hz, 2*J* = 8.0 Hz, 1H), 4.56 (t, 1*J* = 4.0 Hz, 2*J* = 4.0 Hz, 2H), 3.86 (t, 1*J* = 4.0 Hz, 2*J* = 4.0 Hz, 1H), 3.57–3.53 (m, 5H), 3.52–3.48 (m, 5H), 3.45–3.38 (m, 3H), 2.91–2.84 (m, 1H), 2.58 (d, *J* = 16.0 Hz, 1H), 1.99 (s, 1H). ^13^C NMR (100 MHz, DMSO-*d*_6_): δ 173.28, 170.55, 167.75, 146.83, 140.34, 136.66, 132.53, 131.87, 129.86, 126.55, 122.35, 122.28, 119.68, 118.36, 117.85, 115.51, 115.47, 111.13, 109.69, 109.24, 107.28, 105.49, 70.21, 70.15, 70.09, 69.28, 69.09, 67.41, 50.03, 49.01, 42.11, 31.77, 31.44, 22.60. MS (ESI) m/z: 779 [M+H]^+^. Anal. Calcd for C_37_H_37_F_3_N_8_O_8_: C, 57.07; H, 4.79; N, 14.39. Found: C, 57.24; H, 4.66; N, 14.48.

1-(3-(1-(2-(2-(2-(2-((2-(2,6-dioxopiperidin-3-yl)-1,3-dioxoisoindolin-4-yl)amino)ethoxy)ethoxy)ethoxy)ethyl)-1H-1,2,3-triazol-4-yl)phenyl)-3-(4-fluorophenyl)urea (Compound **5e**): Yield 69.9%; ^1^H NMR (400 MHz, DMSO-*d*_6_) δ 11.09 (s, 1H), 8.78 (s, 1H), 8.71 (s, 1H), 8.46 (s, 1H), 8.01 (s, 1H), 7.57–7.53 (m, 1H), 7.50–7.46 (m, 2H), 7.41–7.30 (m, 3H), 7.14–7.07 (m, 3H), 7.02 (d, *J* = 8.0 Hz, 1H), 6.56 (t, 1*J* = 4.0 Hz, 2*J* = 8.0 Hz, 1H), 5.08–5.02 (m, 1H), 4.55 (t, 1*J* = 8.0 Hz, 2*J* = 4.0 Hz, 2H), 3.85 (t, 1*J* = 8.0 Hz, 2*J* = 4.0 Hz, 2H), 3.57–3.47 (m, 11H), 3.42–3.38 (m, 2H), 2.92–2.83 (m, 1H), 2.58 (d, *J* = 16.0 Hz, 1H), 2.04–1.98 (m, 1H). ^13^C NMR (100 MHz, DMSO-*d*_6_) δ 173.28, 170.56, 169.40, 167.76, 158.63, 157.05, 153.10, 146.84, 146.68, 140.69, 136.66, 136.46, 132.54, 131.82, 129.81, 122.23, 120.52, 120.47, 119.33, 118.14, 117.86, 115.82, 115.67, 115.27, 111.13, 109.70, 70.22, 70.15, 70.10, 69.29, 69.10, 50.03, 49.02, 42.12, 40.53, 31.45, 22.61. MS (ESI) m/z: 729 [M+H]^+^. Anal. Calcd for C_36_H_37_FN_8_O_8_: C, 59.33; H, 5.12; N, 15.38. Found: C, 59.16; H, 5.10; N, 15.29.

1-(4-(1-(2-(2-(2-(2-((2-(2,6-dioxopiperidin-3-yl)-1,3-dioxoisoindolin-4-yl)amino)ethoxy)ethoxy)ethoxy)ethyl)-1H-1,2,3-triazol-4-yl)phenyl)-3-(4-fluorophenyl)urea (Compound **6a**): Yield 69.2%; ^1^H NMR (400 MHz, DMSO-*d*_6_) δ 11.10 (s, 1H), 8.78 (s, 1H), 8.74 (s, 1H), 8.41 (s, 1H), 7.75 (d, *J* = 4.0 Hz, 1H), 7.56 (t, 1*J* = 4.0 Hz, 2*J* = 4.0 Hz, 1H), 7.53 (d, *J* = 4.0 Hz, 2H), 7.49–7.46 (m, 2H), 7.15–7.09 (m, 3H), 7.03 (d, *J* = 8.0 Hz, 1H), 6.57 (t, 1*J* = 4.0 Hz, 2*J* = 4.0 Hz, 1H), 5.05 (dd, 1*J* = 4.0 Hz, 2*J* = 4.0 Hz, 1H), 4.54 (t, 1*J* = 8.0 Hz, 2*J* = 4.0 Hz, 2H), 3.85 (t, 1*J* = 4.0 Hz, 2*J* = 4.0 Hz, 2H), 3.58–3.50 (m, 11H), 3.42 (dd, 1*J* = 4.0 Hz, 2*J* = 4.0 Hz, 2H), 2.91–2.85 (m, 1H), 2.58 (d, *J* = 8.0 Hz, 1H), 2.06–1.98 (m, 1H). ^13^C NMR (100 MHz, DMSO-*d*_6_) δ 173.30, 170.56, 169.40, 167.76, 158.64, 157.06, 157.03, 153.03, 146.84, 146.66, 143.13, 139.80, 136.67, 136.43, 132.54, 126.16, 124.99, 121.36, 120.51, 120.46, 118.91, 117.86, 115.83, 115.69, 111.14, 109.70, 70.22, 70.16, 70.11, 69.30, 69.14, 50.02, 49.02, 42.13, 31.45, 22.60. MS (ESI) m/z: 729 [M+H]^+^. Anal. Calcd for C_36_H_37_FN_8_O_8_: C, 59.33; H, 5.12; N, 15.38. Found: C, 59.42; H, 5.07; N, 15.24.

1-(4-cyanophenyl)-3-(4-(1-(2-(2-(2-(2-((2-(2,6-dioxopiperidin-3-yl)-1,3-dioxoisoindolin-4-yl)amino)ethoxy)ethoxy)ethoxy)ethyl)-1H-1,2,3-triazol-4-yl)phenyl)urea (Compound **6b**): Yield 55.8%; ^1^H NMR (400 MHz, DMSO-*d*_6_) δ 11.10 (s, 1H), 9.28 (s, 1H), 9.01 (s, 1H), 8.42 (s, 1H), 7.76 (dd, 1*J* = 4.0 Hz, *2J* = 8.0 Hz, 1H), 7.66 (d, *J* = 4.0 Hz, 2H), 7.57–7.54 (m, 3H), 7.10 (d, *J* = 4.0 Hz, 1H), 7.03 (d, *J* = 4.0 Hz, 1H), 6.57 (t, 1*J* = 4.0 Hz, 2*J* = 4.0 Hz, 1H), 5.07–5.04 (m, 1H), 4.54(t, 1*J* = 8.0 Hz, 2*J* = 4.0 Hz, 2H), 3.85 (t, 1*J* = 4.0 Hz, 2*J* = 4.0 Hz, 2H), 3.55–3.50 (m, 8H), 3.42 (t, 1*J* = 4.0 Hz, 2*J* = 4.0 Hz, 2H), 2.91–2.85 (m, 1H), 2.58 (d, *J* = 12.0 Hz, 2H), 2.03–2.01 (m, 1H). ^13^C NMR (100 MHz, DMSO-*d*_6_) δ 173.30, 170.56, 169.40, 167.76, 152.51, 146.84, 146.57, 144.65, 139.25, 136.67, 133.77, 132.53, 126.19, 125.49, 121.48, 119.80, 119.45, 119.23, 119.05, 118.54, 117.86, 111.14, 109.70, 103.74, 70.22, 70.15, 70.11, 69.30, 69.14, 50.04, 49.02, 42.13, 40.50, 31.45, 22.60. MS (ESI) m/z: 736 [M+H]^+^. Anal. Calcd for C_37_H_37_N_9_O_8_: C, 60.40; H, 5.07; N, 17.13. Found: C, 60.21; H, 5.01; N, 17.25.

1-(4-bromophenyl)-3-(4-(1-(2-(2-(2-(2-((2-(2,6-dioxopiperidin-3-yl)-1,3-dioxoisoindolin-4-yl)amino)ethoxy)ethoxy)ethoxy)ethyl)-1H-1,2,3-triazol-4-yl)phenyl)urea (Compound **6c**): Yield 74.9%; ^1^H NMR (400 MHz, DMSO-*d*_6_) δ 11.10 (s, 1H), 8.84 (s, 1H), 8.81 (s, 1H), 8.41 (s, 1H), 7.75 (d, *J* = 8.0 Hz, 2H), 7.57–7.52 (m, 3H), 7.47–7.43 (m, 5H), 7.11 (d, *J* = 4.0 Hz, 1H), 7.04 (d, *J* = 4.0 Hz, 1H), 6.58 (t, 1*J* = 4.0 Hz, 2*J* = 4.0 Hz, 1H), 5.07–5.04 (m, 1H), 4.54 (t, 1*J* = 4.0 Hz, 2*J* = 4.0 Hz, 2H), 3.85 (t, 1*J* = 4.0 Hz, 2*J* = 4.0 Hz, 2H), 3.58–3.51 (m, 10H), 3.43–3.41 (m, 2H), 2.91–2.85 (m, 1H), 2.60–2.55 (m, 1H), 2.04–2.01 (m, 1H). ^13^C NMR (100 MHz, DMSO-*d*_6_) δ 173.29, 170.95, 170.56, 169.41, 167.76, 165.81, 152.80, 146.85, 146.63, 139.61, 139.56, 136.67, 132.55, 132.00, 126.17, 125.15, 121.40, 120.72, 120.64, 119.00, 117.87, 113.73, 111.14, 109.71, 70.23, 70.16, 70.11, 69.31, 69.14, 50.02, 49.02, 42.13, 31.45, 22.60. MS (ESI) m/z: 789 [M+H]^+^. Anal. Calcd for C_36_H_37_BrN_8_O_8_: C, 54.76; H, 4.72; N, 14.19. Found: C, 54.63; H, 4.77; N, 14.27.

1-(4-chlorophenyl)-3-(4-(1-(2-(2-(2-(2-((2-(2,6-dioxopiperidin-3-yl)-1,3-dioxoisoindolin-4-yl)amino)ethoxy)ethoxy)ethoxy)ethyl)-1H-1,2,3-triazol-4-yl)phenyl)urea (Compound **6d**): Yield 70.04%; ^1^H NMR (400 MHz, DMSO-*d*_6_) δ 11.10 (s, 1H), 8.83 (s, 1H), 8.80 (s, 1H), 8.41 (s, 1H), 7.75 (d, *J* = 8.0 Hz, 2H), 7.57–7.49 (m, 5H), 7.34 (d, *J* = 4.0 Hz, 2H), 7.11 (d, *J* = 4.0 Hz, 1H), 7.03 (d, *J* = 8.0 Hz, 1H), 6.57(t, 1*J* = 4.0 Hz, 2*J* = 4.0 Hz, 1H), 5.07–5.04 (m, 1H), 4.54 (t, 1*J* = 4.0 Hz, 2*J* = 4.0 Hz, 2H), 3.85 (t, 1*J* = 4.0 Hz, 2*J* = 4.0 Hz, 2H), 3.58–3.50 (m, 10H), 3.42 (dd, 1*J* = 4.0 Hz, 2*J* = 4.0 Hz, 2H), 2.91–2.85 (m, 1H), 2.60–2.57 (m, 1H), 2.03–2.01 (m, 1H). ^13^C NMR (100 MHz, DMSO-*d*_6_) δ 173.30, 173.26, 170.57, 167.77, 152.86, 146.85, 146.66, 139.71, 139.18, 136.68, 132.53, 129.09, 126.17, 125.85, 125.06, 121.42, 120.32, 120.25, 119.00, 111.15, 105.91, 91.79, 88.37, 75.29, 73.74, 70.22, 70.16, 70.11, 69.30, 69.14, 50.05, 49.02, 42.13, 31.76, 31.45, 22.61. MS (ESI) m/z: 745 [M+H]^+^. Anal. Calcd for C_36_H_37_ClN_8_O_8_: C, 58.02; H, 5.00; N, 15.04. Found: C, 58.23; H, 4.94; N, 15.07.

1-(4-(1-(2-(2-(2-(2-((2-(2,6-dioxopiperidin-3-yl)-1,3-dioxoisoindolin-4-yl)amino)ethoxy)ethoxy)ethoxy)ethyl)-1H-1,2,3-triazol-4-yl)phenyl)-3-(4-(trifluoromethyl)phenyl)urea (Compound **6e**): Yield 71.92%; ^1^H NMR (400 MHz, DMSO-*d*_6_) δ 11.10 (s, 1H), 9.86–9.70 (m, 1H), 8.42 (s, 1H), 7.76 (d, *J* = 4.0 Hz, 2H), 7.69 (d, *J* = 4.0 Hz, 2H), 7.64 (d, *J* = 4.0 Hz, 2H), 7.58–7.54 (m, 3H), 7.11 (d, *J* = 4.0 Hz, 1H), 7.03 (d, *J* = 8.0 Hz, 1H), 6.57 (t, 1*J* = 4.0 Hz, 2*J* = 4.0 Hz, 1H), 5.07–5.04 (m, 1H), 4.54 (t, 1*J* = 4.0 Hz, 2*J* = 4.0 Hz, 2H), 3.85 (t, 1*J* = 4.0 Hz, 2*J* = 4.0 Hz, 2H), 3.58–3.50 (m, 12H), 2.90–2.85 (m, 1H), 2.63–2.57 (m, 1H), 2.04–1.98 (m, 1H), 1.88–1.80 (m, 1H). ^13^C NMR (100 MHz, DMSO-*d*_6_): δ 174.30, 172.56, 169.30, 168.76, 152.31, 147.84, 145.57, 144.25, 139.55, 137.67, 134.77, 132.53, 128.87, 126.19, 125.49, 121.48, 119.80, 119.45, 119.23, 119.05, 118.84, 118.54, 117.86, 117.05, 111.14, 109.70, 103.74, 70.22, 70.15, 70.11, 69.30, 69.14, 50.04, 49.02, 42.13, 40.50, 31.45, 21.60. MS (ESI) m/z: 779 [M+H]^+^. Anal. Calcd for C_37_H_37_F_3_N_8_O_8_: C, 57.07; H, 4.79; N, 14.39. Found: C, 57.18; H, 4.59; N, 14.53.

### 3.5. Cell Culture

Human breast cancer cell line MCF-7 and human hepatocellular carcinoma cell line Huh7 were cultured in DMEM medium containing 10% FBS and 1% penicillin/streptomycin. Cells were maintained in a humidified incubator (5% CO_2_/95% air atmosphere at 37 °C).

### 3.6. Cell Viability Assay

Cell viability was evaluated by CCK-8 assay according to the manufacturer’s instructions. Cells were seeded in a 96-well plate at the density of 5 × 10^3^ cells per well. After incubation for 24 h, cells were treated with different doses (0, 5 μM, 10 μM, 20 μM) of new synthetic compounds and cultured for 48 h. Then, CCK-8 reagent was added to each well for one hour of incubation. Absorbance was measured using a Microplate Reader at the wavelength of 450 nm. For IC_50_, cells were treated with different concentrations of compounds (0, 10, 20, 40, 80, 160 μM) for 48 h and cell viability was measured. Then, the inhibition percentage was calculated and the IC_50_ was analyzed using the prism statistical software.

### 3.7. Live and Dead Cells Assay

Cells were seeded in a 96-well plate with the density of 5 × 10^3^ cells per well. Different concentrations (0, 5 μM, 10 μM, 20 μM) of compounds were added to cells and cultured for 24 h. Cells were then stained using Calcein/PI Live/Dead Viability Assay Kit, the living cells were stained green and dead cells were stained red. Live and dead cells numbers were observed and photographed using a fluorescent confocal microscope.

### 3.8. Plate Clone Formation Assay

Cells were seeded in a 6-well plate at a density of 500 cells per well. After adhesion, cells were treated with different doses of compounds (0, 5 μM, 10 μM, 20 μM) for 48 h. Then, cells were cultured continually for about 10 days until eye visible monoclonal cells appeared. Cells were then fixed with 4% paraformaldehyde and stained using Giemsa dye. Clone numbers were photographed and counted using an optical microscope.

### 3.9. ROS Measurement

Cells were seeded in a 96-well plate with the density of 5 × 10^3^ cells per well. Different doses of compounds (0, 5 μM, 10 μM, 20 μM) were added to cells for 24 h. After treatment, cells were stimulated with 10 μM DCFH-DA for 30 min at 37 °C. A fluorescent confocal microscope was used to observe and photograph the cellular ROS levels.

### 3.10. Apoptosis Measurement

Annexin V-FITC/PI apoptosis detection was performed according to the manufacturer’s instruction. Cells were cultured in a 6-well plate and treated with different concentrations of compounds (0, 5 μM, 10 μM, 20 μM) for 48 h. Then, cells were collected and washed with PBS and suspended in Annexin V binding buffer. FITC labeled Annexin V and PI were added and incubated for 20 min in the dark. Samples were measured using flow cytometry as soon as possible and the raw data was analyzed using FlowJo software (v10, Becton, Dickinson and Company, Ashland, CA, USA).

### 3.11. Western Blot

Protein expressions were evaluated by western blot. Cells were cultured in a 12-well plate and treated with different concentrations of compounds (0, 5 μM, 10 μM, 20 μM) for 48 h. Then cells proteins were collected using radioimmunoprecipitation assay (RIPA) buffer containing protease/phosphatase inhibitor cocktail. Proteins were separated and collected using a 10–12% sodium dodecyl sulfate polyacrylamide gel electrophoresis and nitrocellulose membranes. After being incubated with antibody, proteins were measured and calculated using the electronic chemiluminescence substrate kit.

### 3.12. Statistical Analyses

Data were presented as means ± SEM and performed using Graph Prim 7.0. A two-tailed Student’s t-test or one-way analysis of variance following Student–Newman–Keuls (SNK) test were used to assess significant differences. Values of *p* < 0.05 were considered statistically significant.

## 4. Conclusions

In summary, we designed and synthesized a series of novel pomalidomide derivatives containing urea moieties. These new compounds were evaluated for their anti-tumor functions in cancer cells lines. The results found that these new compounds exhibited more effective anti-tumor ability in MCF-7 cells than in Huh7 cells. Particularly, compound **5d** showed the most promising effects on cancer cells, with IC_50_ values of 20.2 μM in MCF-7. Moreover, studies demonstrated that compound **5d** inhibited cell proliferation, increased cellular ROS levels, and induced DNA damage, leading to cell apoptosis and cell death. These observations may highlight the potential anti-tumor effects of these novel compounds and will provide novel therapy for cancer treatment.

## Figures and Tables

**Table 1:** Cell toxicity and viability of compounds **5a**–**5e** and **6a**–**6e**.

Compd No.	IC_50_ (μM)	
	MCF-7	Huh7
**5a**	86.57 ± 8.59	>200
**5b**	114.13 ± 6.91	>200
**5c**	26.93 ± 2.40	132.27 ± 2.18
**5d**	20.2 ± 5.70	>200
**5e**	53.44 ± 0.49	>200
**6a**	>200	>200
**6b**	>200	>200
**6c**	150.73 ± 5.90	>200
**6d**	107.60 ± 13.17	>200
**6e**	99.14 ± 9.51	>200
con1	>200	>200
con2	>200	>200
con3	>200	>200

## Data Availability

Data is contained within the article and supplementary material.

## Supplementary Materials

The following supporting information can be downloaded at: https://www.mdpi.com/article/10.3390/ph15121479/s1, Figures S1–S10: ^1^H NMR spectrum (400MHz, DMSO-*d*_6_) and ^13^C NMR spectrum (100MHz, DMSO-*d*_6_) spectrum of compound **5a**, **5b**, **5c**, **5d**, **5e**, **6a**, **6b**, **6c**, **6d**, **6e**.
