# Supplementary material for: Synthesis and Anti-Tumor Effects of Novel Pomalidomide Derivatives Containing Urea Moieties
Source: Pharmaceuticals (Basel). 2022 Nov 27;15(12):1479. doi: 10.3390/ph15121479 (PMC9785895; doi:10.3390/ph15121479)

Figure S1. ^{1}H NMR (400MHz, DMSO-d_6) and ^{13}C NMR (100MHz, DMSO-d_6) spectrum of compound 5a

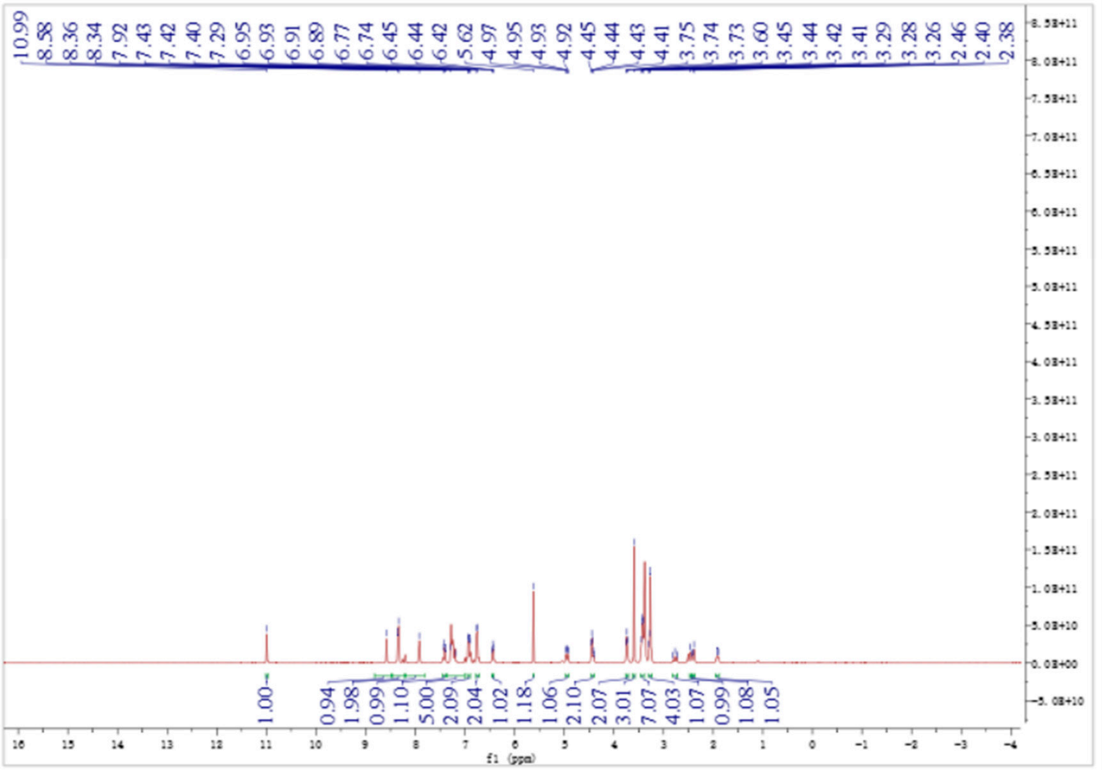

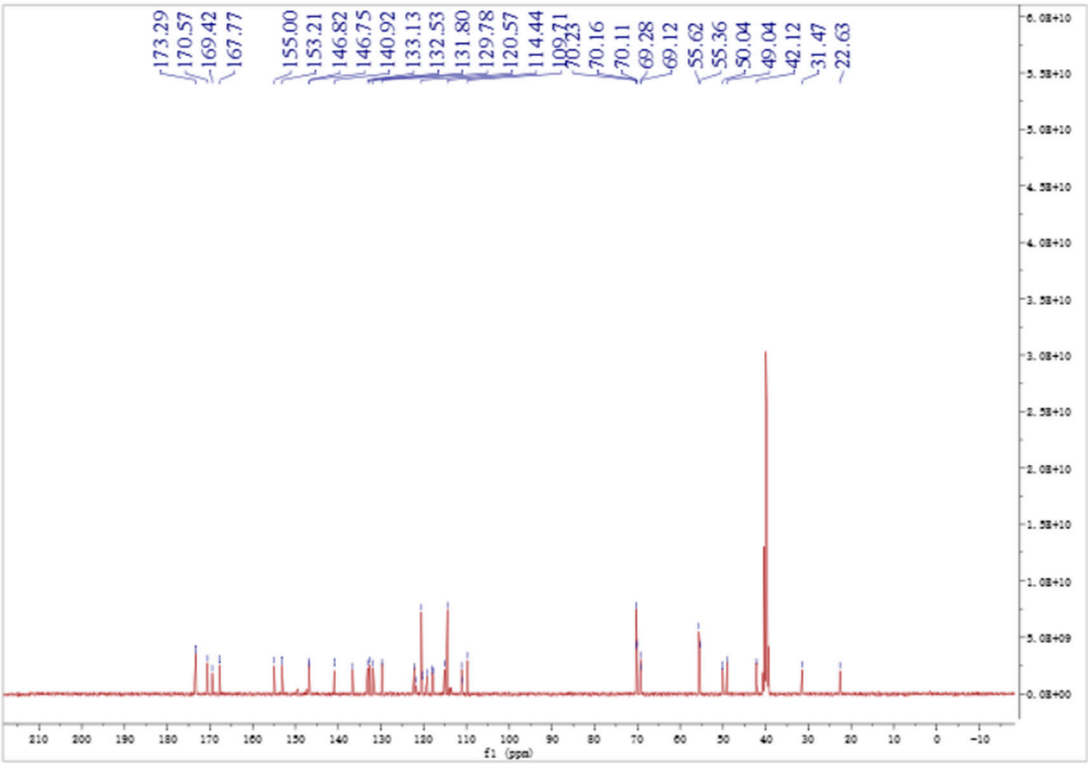

Figure S2. ^{1}H NMR (400MHz, DMSO-*d*6) and ^{13}C NMR (100MHz, DMSO-*d*6) spectrum of compound 5b

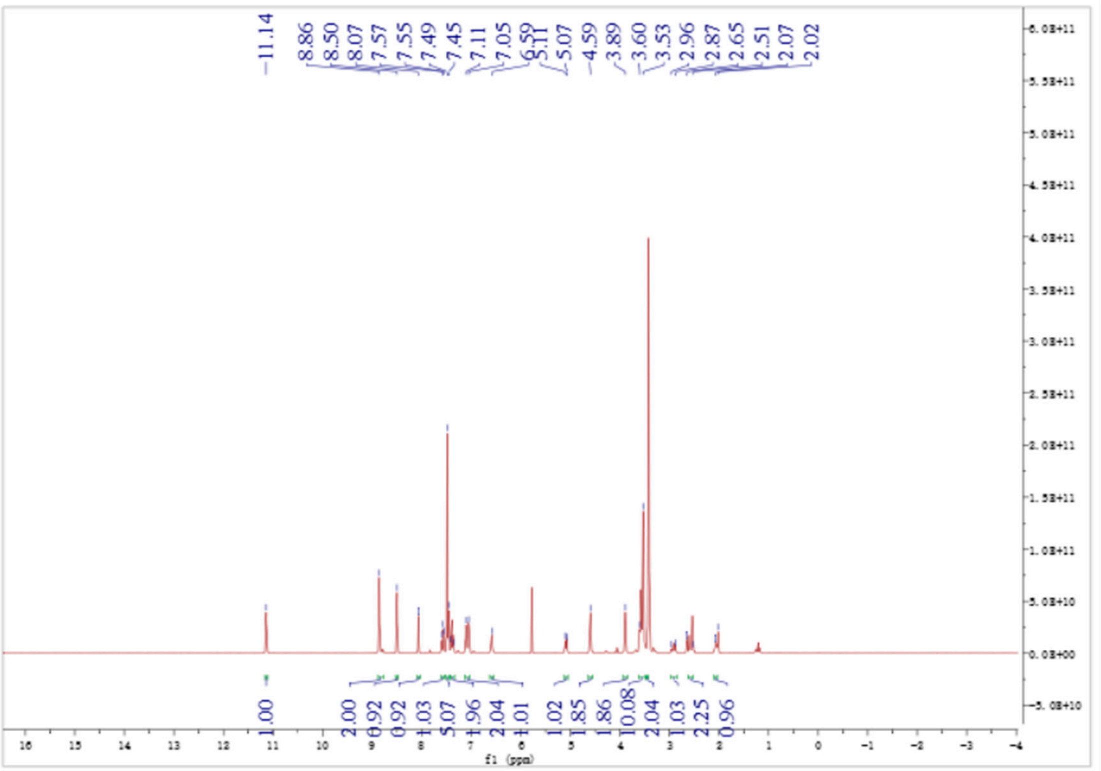

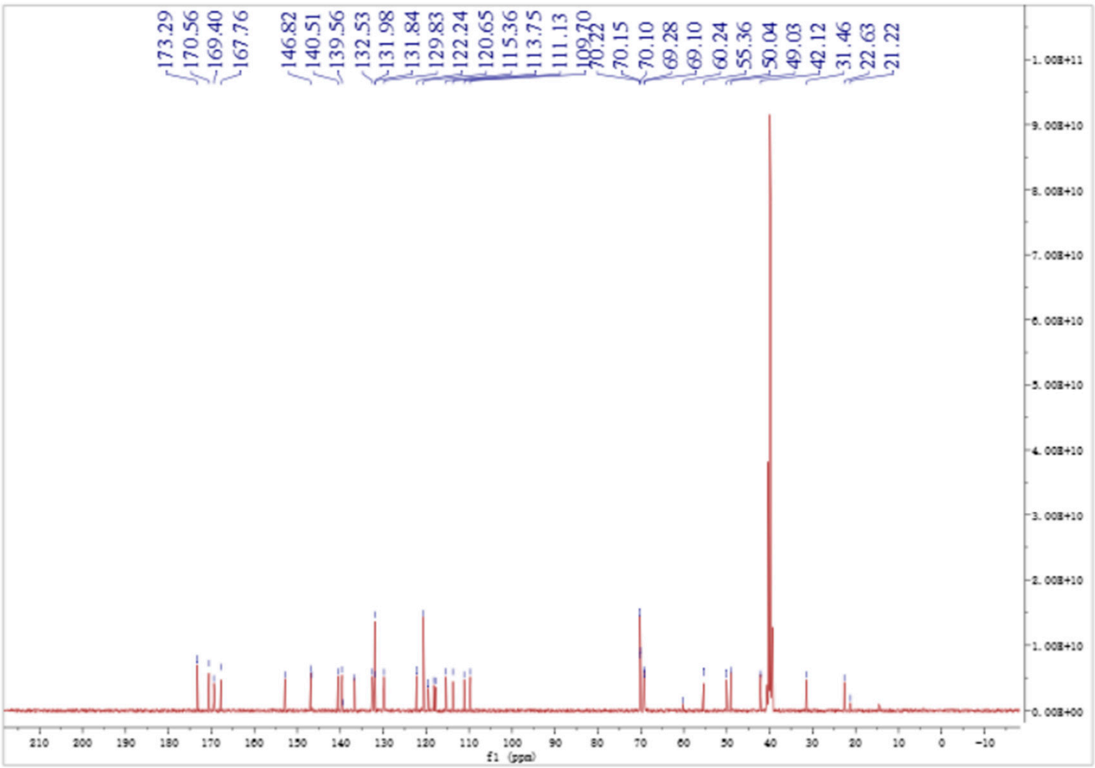

Figure S3. ^{1}H NMR (400MHz, DMSO-d_6) and ^{13}C NMR (100MHz, DMSO-d_6) spectrum of compound 5c

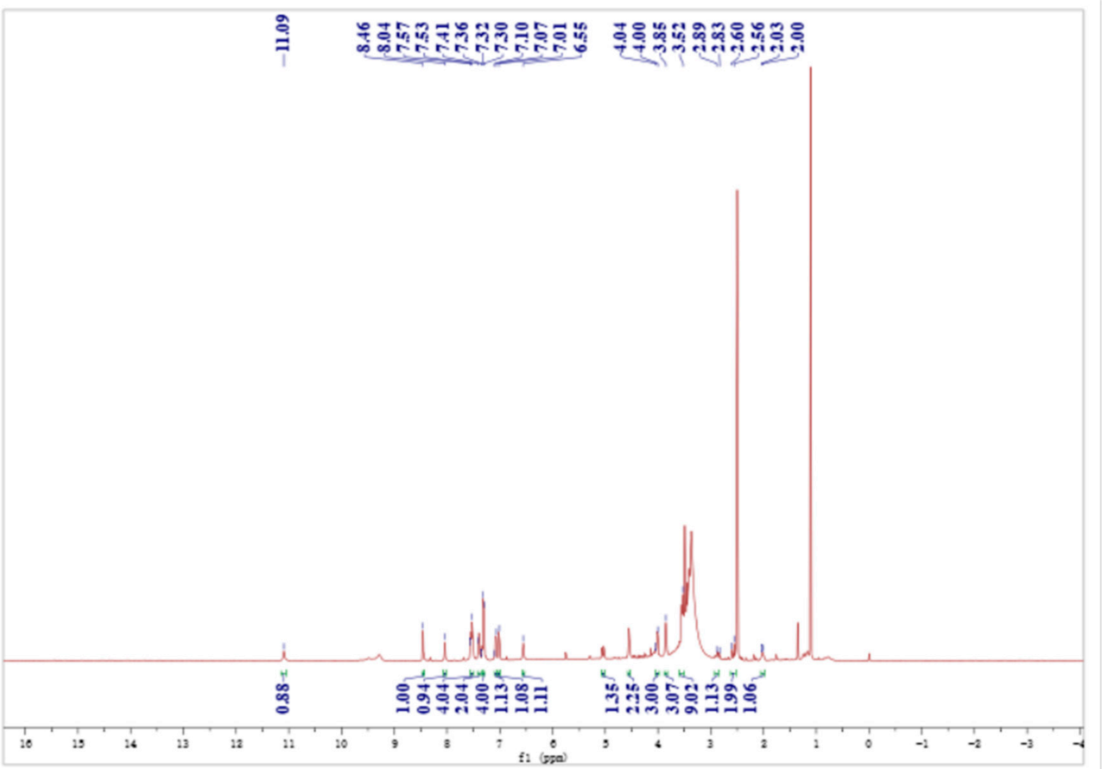

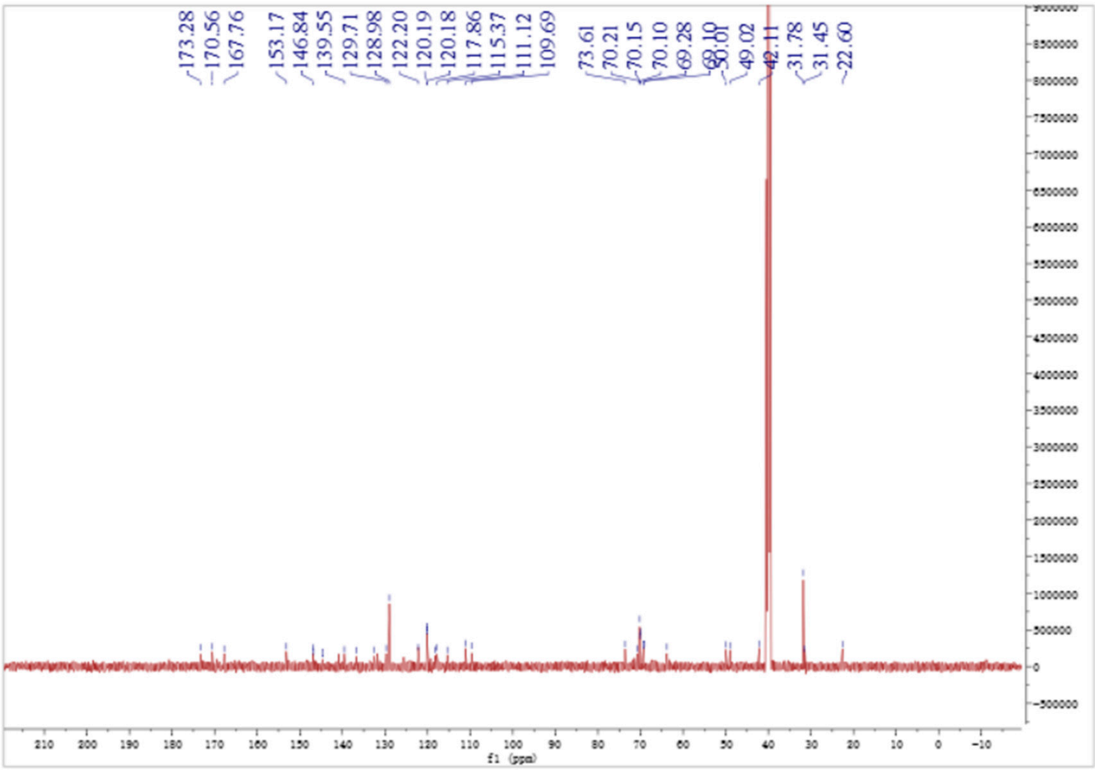

Figure S4. ^{1}H NMR (400MHz, DMSO-*d*6) and ^{13}C NMR (100MHz, DMSO-*d*6) spectrum of compound 5d

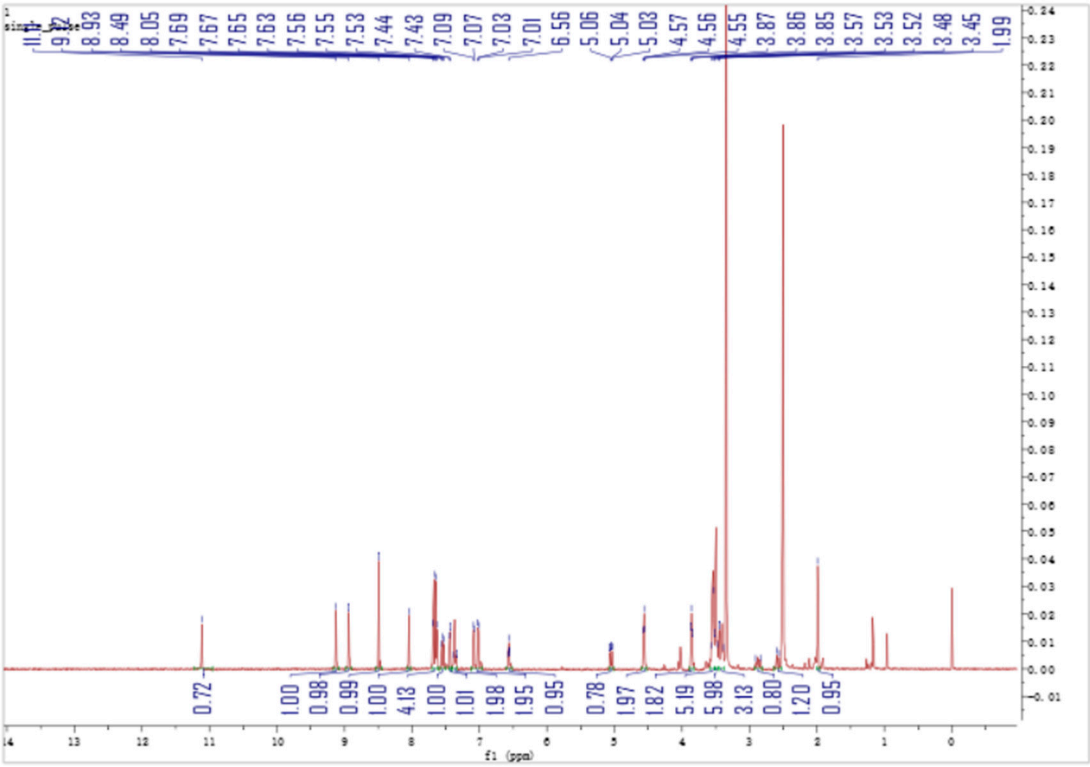

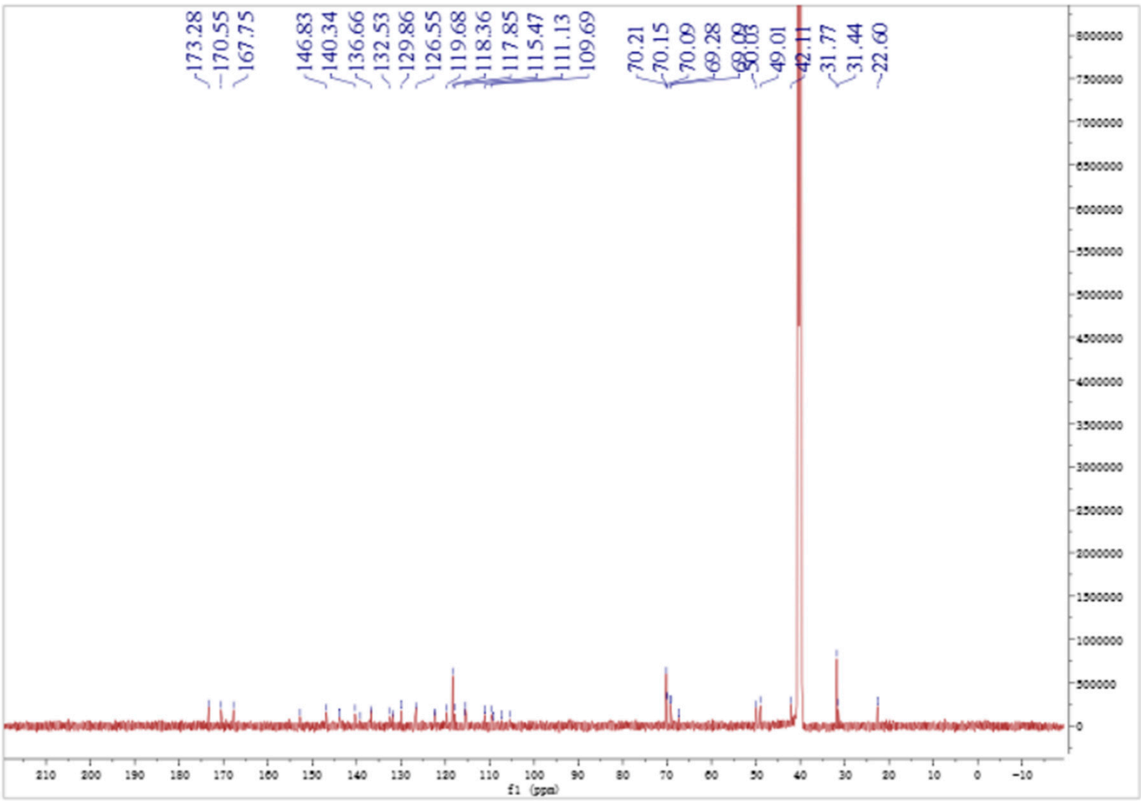

Figure S5. ^{1}H NMR (400MHz, DMSO-d_6) and ^{13}C NMR (100MHz, DMSO-d_6) spectrum of compound 5e

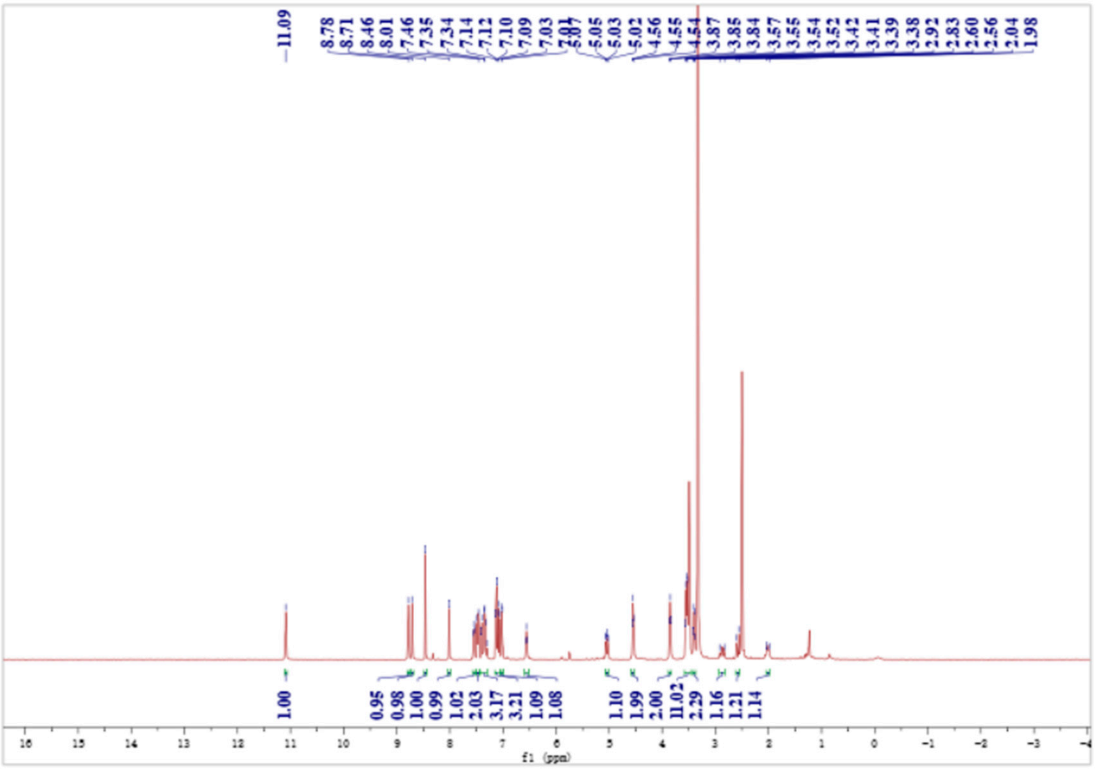

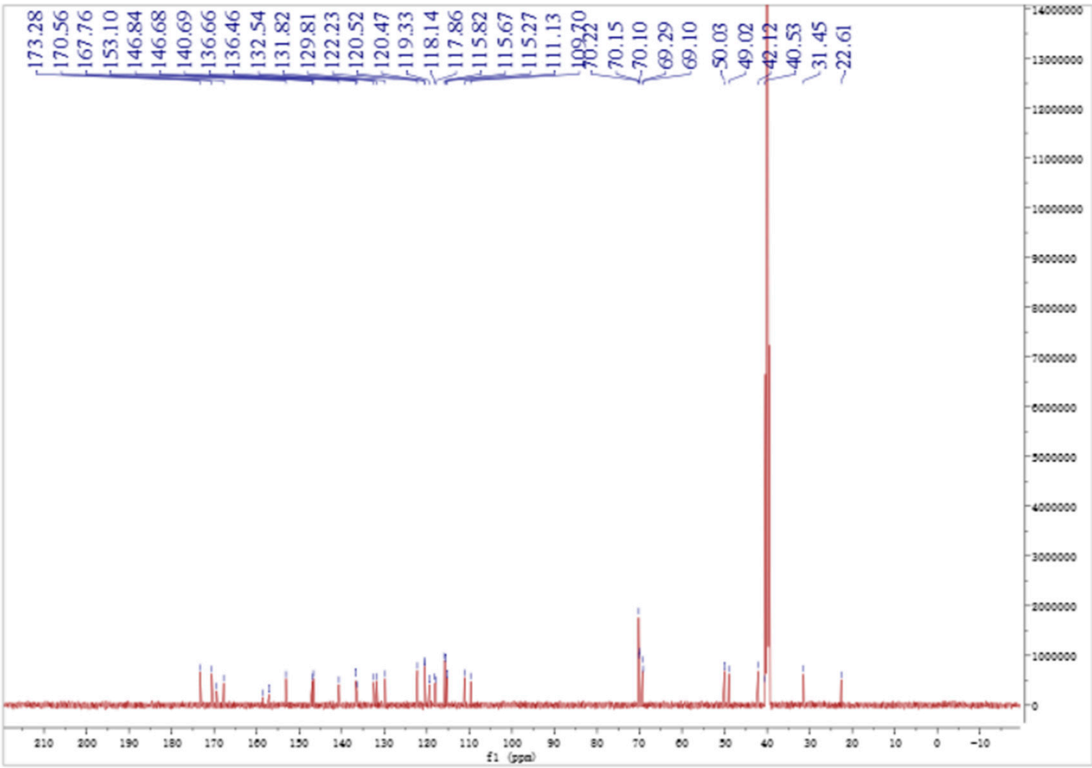

Figure S6. ^{1}H NMR (400MHz, DMSO-d_6) and ^{13}C NMR (100MHz, DMSO-d_6) spectrum of compound 6a

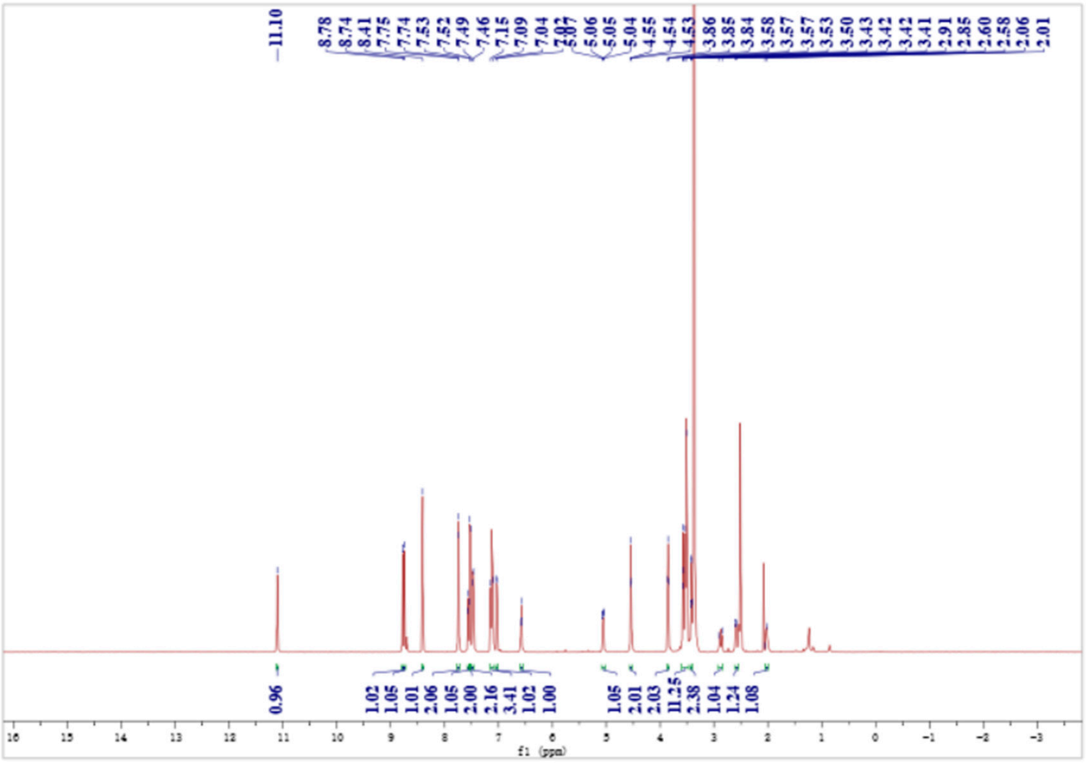

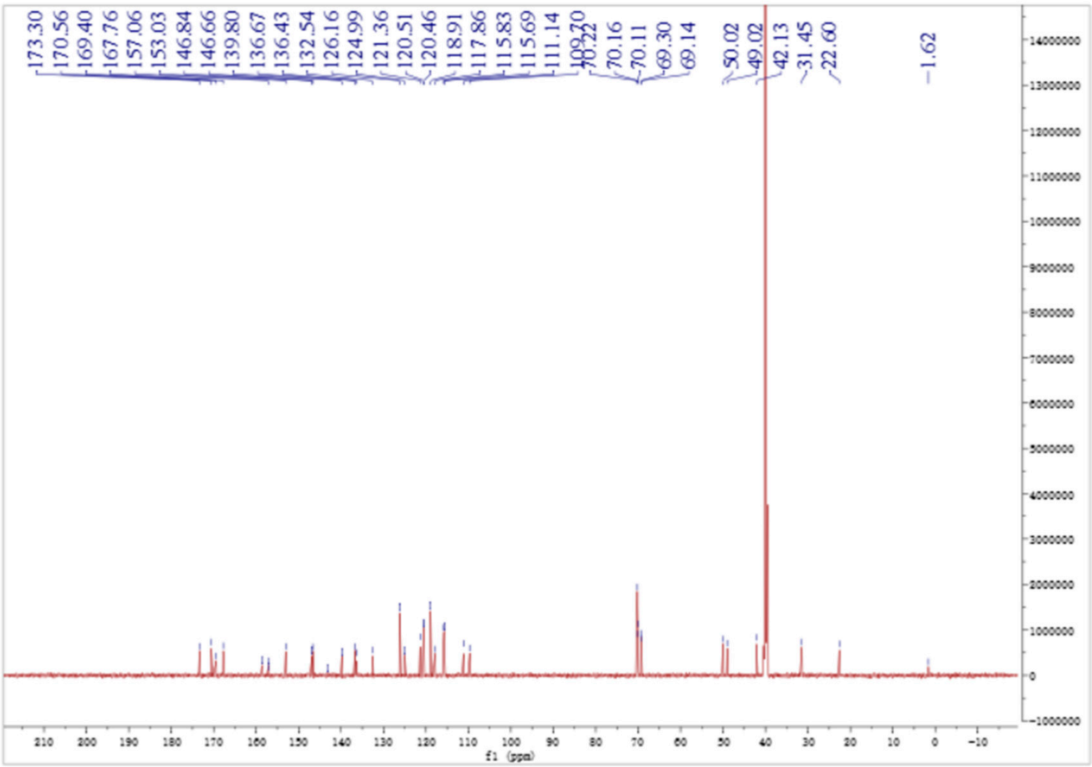

Figure S7. ^{1}H NMR (400MHz, DMSO-d_6) and ^{13}C NMR (100MHz, DMSO-d_6) spectrum of compound 6b

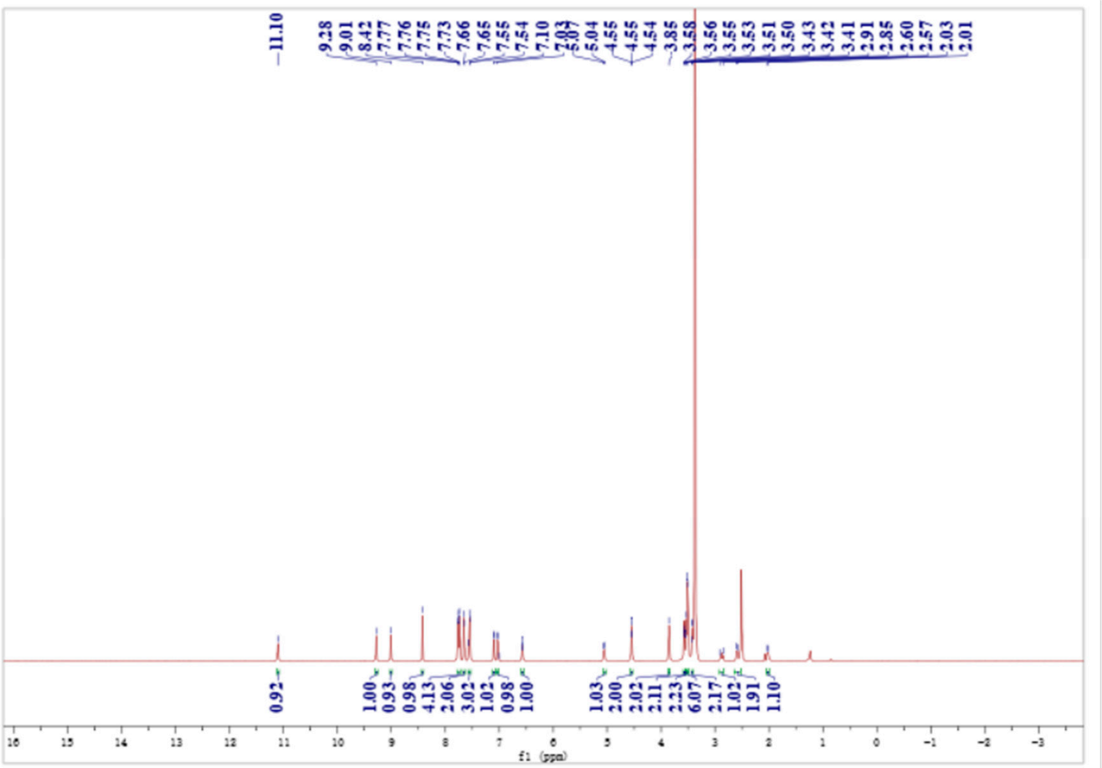

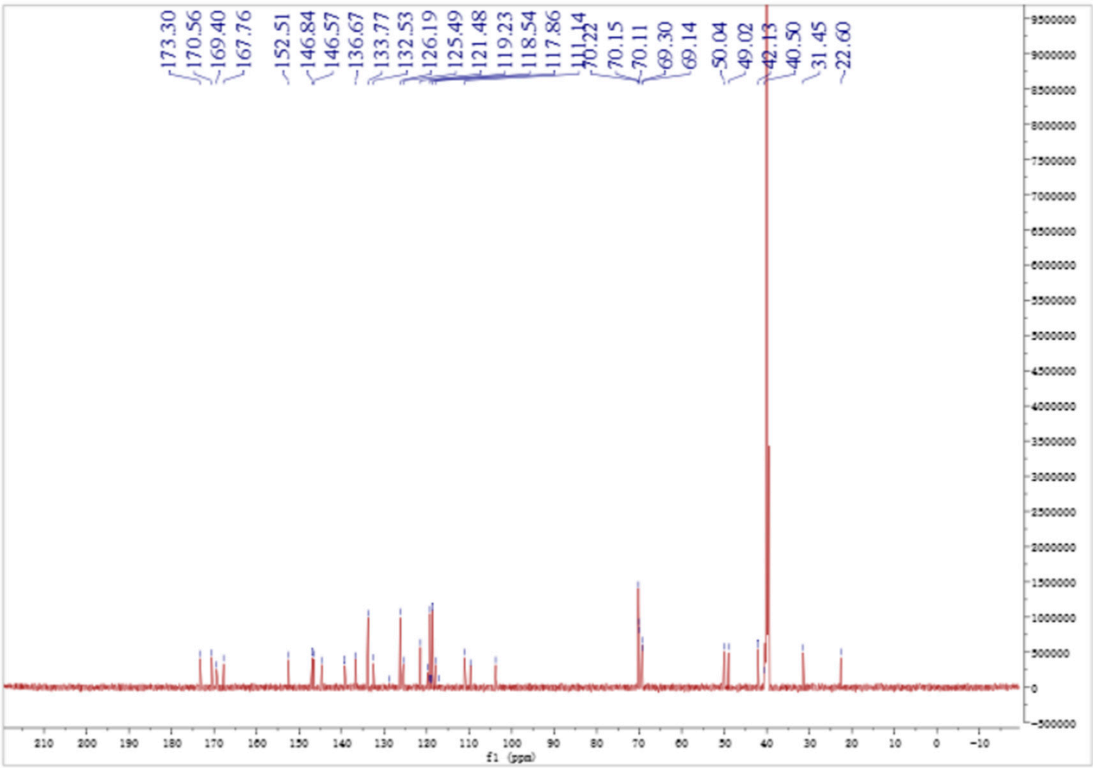

Figure S8. ^{1}H NMR (400MHz, DMSO-d_6) and ^{13}C NMR (100MHz, DMSO-d_6) spectrum of compound 6c

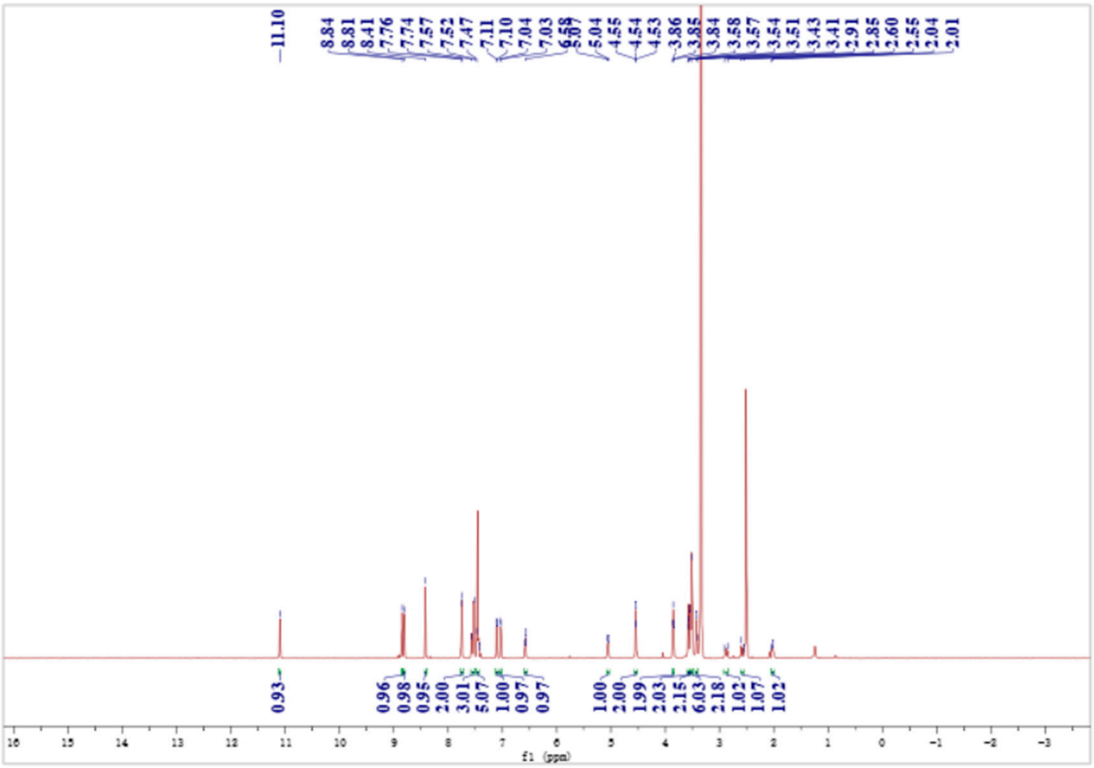

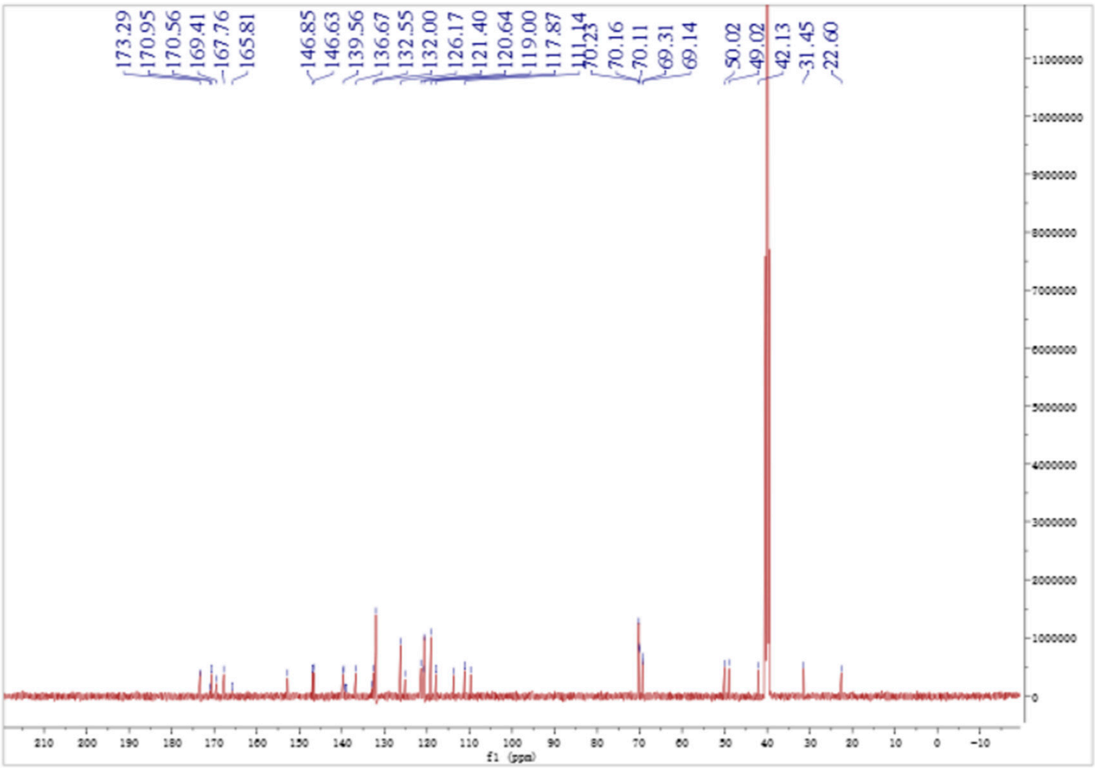

Figure S9. ^{1}H NMR (400MHz, DMSO-d_6) and ^{13}C NMR (100MHz, DMSO-d_6) spectrum of compound 6d

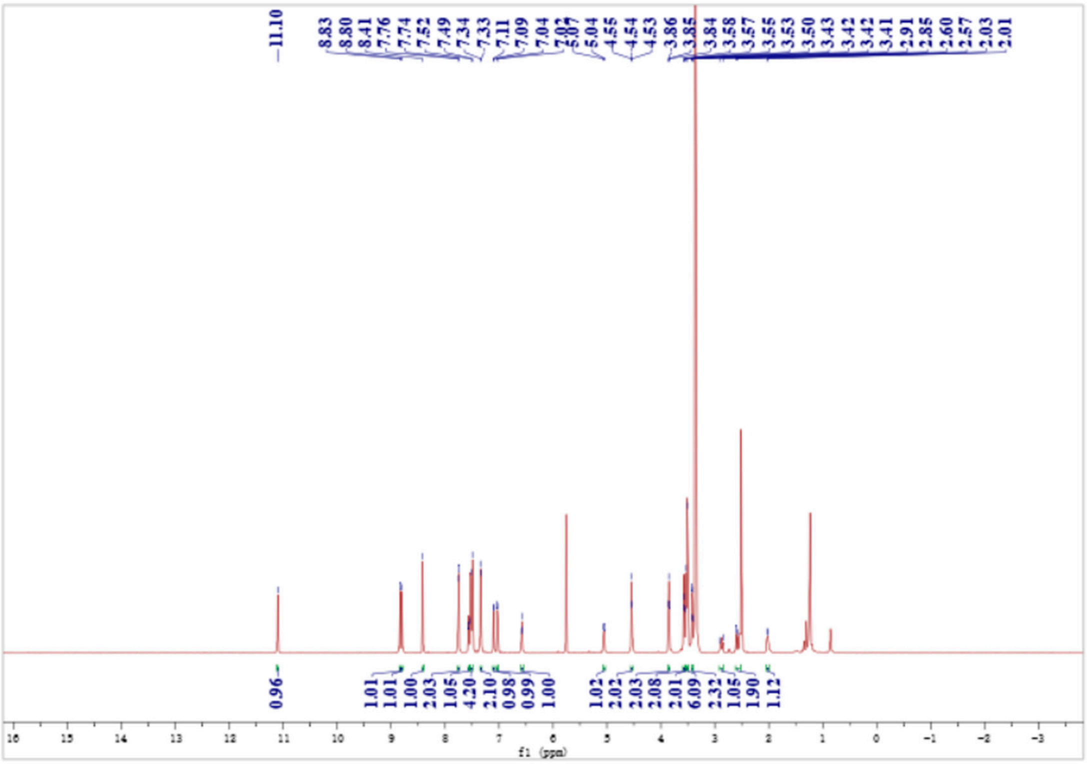

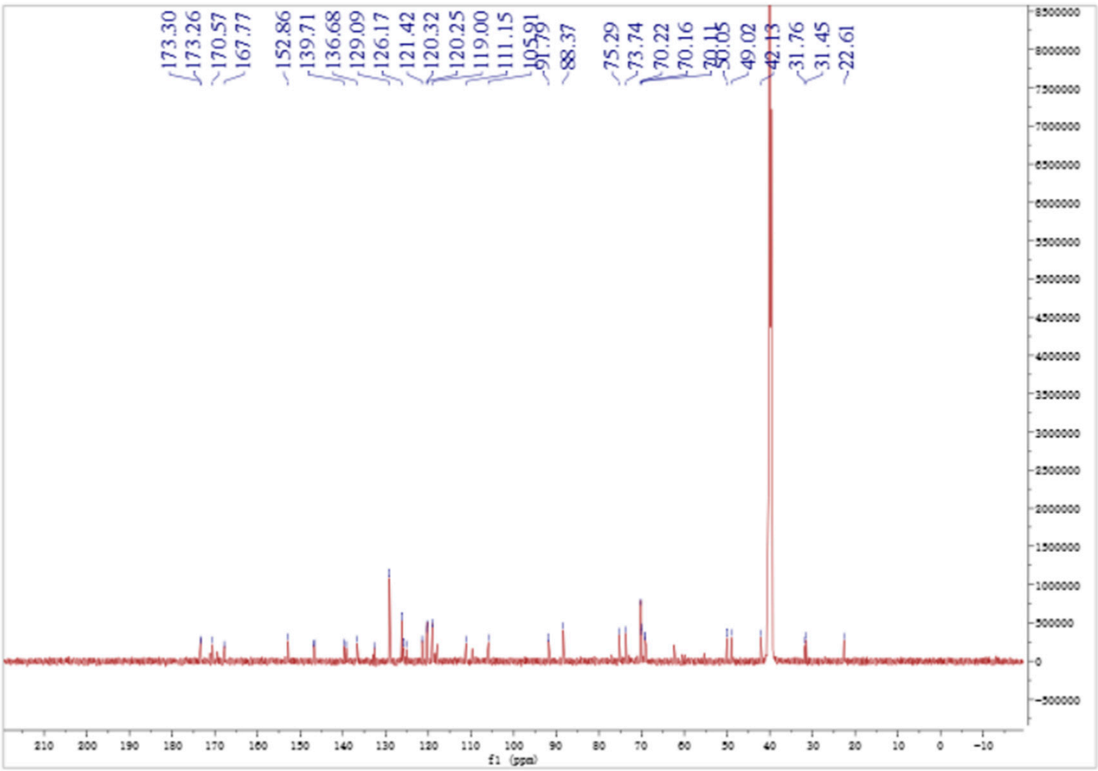

Figure S10. ^{1}H NMR (400MHz, DMSO-d_6) spectrum of compound 6e

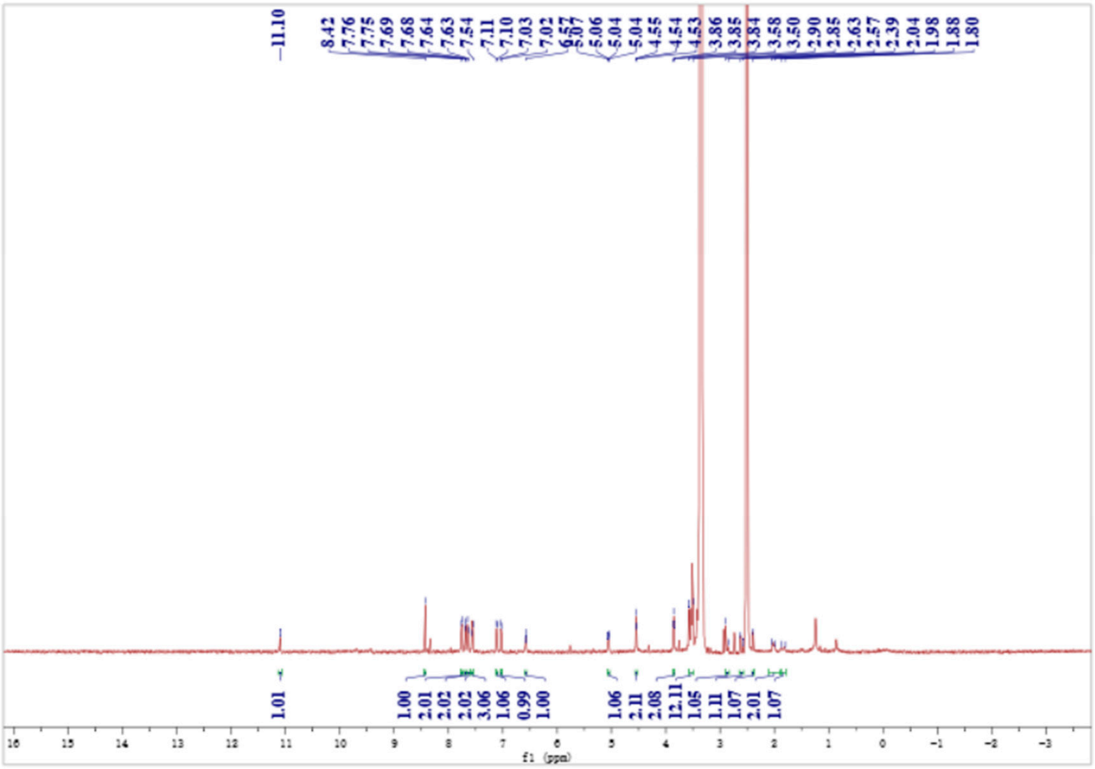

Supplement: Supplementary file 1 [file pharmaceuticals-15-01479-s001.zip › Supplementary Materials.pdf]
